# Structural Properties of Gene Promoters Highlight More than Two Phenotypes of Diabetes

**DOI:** 10.1371/journal.pone.0137950

**Published:** 2015-09-17

**Authors:** Constantin Ionescu-Tîrgovişte, Paul Aurelian Gagniuc, Cristian Guja

**Affiliations:** 1 National Institute of Diabetes, Nutrition and Metabolic Diseases “N.C. Paulescu”, Bucharest, Romania; 2 National Institute of Pathology "Victor Babes", Bucharest, Romania; 3 Department of Genetics, University of Bucharest, Aleea Portocalelor 1–3, Sector 6, Bucharest, Romania; Université Paris Descartes, FRANCE

## Abstract

Genome-wide association studies (GWAS) published in the last decade raised the number of loci associated with type 1 (T1D) and type 2 diabetes (T2D) to more than 50 for each of these diabetes phenotypes. The environmental factors seem to play an important role in the expression of these genes, acting through transcription factors that bind to promoters. Using the available databases we examined the promoters of various genes classically associated with the two main diabetes phenotypes. Our comparative analyses have revealed significant architectural differences between promoters of genes classically associated with T1D and T2D. Nevertheless, five gene promoters (about 16%) belonging to T1D and six gene promoters (over 19%) belonging to T2D have shown some intermediary structural properties, suggesting a direct relationship to either LADA (Latent Autoimmune Diabetes in Adults) phenotype or to non-autoimmune type 1 phenotype. The distribution of these promoters in at least three separate classes seems to indicate specific pathogenic pathways. The image-based patterns (DNA patterns) generated by promoters of genes associated with these three phenotypes support the clinical observation of a smooth link between specific cases of typical T1D and T2D. In addition, a global distribution of these DNA patterns suggests that promoters of genes associated with T1D appear to be evolutionary more conserved than those associated with T2D. Though, the image based patterns obtained by our method might be a new useful parameter for understanding the pathogenetic mechanism and the diabetogenic gene networks.

## Introduction

Diabetes mellitus is a heterogeneous syndrome with an onset that can occur from birth to any point in one's lifetime [1]. The hereditary nature of diabetes is long known, but its genetic basis started to be unravelled only in the 7th decade of the last century [2,3]. It was found that the common phenotypes of diabetes are polygenic and not monogenic, as previously supposed according to the Mendel laws of hereditary. It is not surprising that ~30 monogenic forms of diabetes could be relatively easy identified [4]. Each of these forms have a different clinical phenotype and, frequently, different therapeutic indications [5]. However, their prevalence does not reach ~5% of the total diabetes cases. The gene-sequencing chips using targeted next-generation sequencing allows their quick and efficient detection [5,6].

The identification of the genetic basis for the common polygenic diabetes phenotypes proved to be a much more difficult issue. A deterrent for this was represented by the incoherence of the diabetes classifications over time. Characterization of diabetes phenotypes begun 150 years ago when Etienne Lancereaux (1829–1910), based only on clinical observation corroborated with forensic studies, reached the conclusion that diabetes is not a simple disease but a complex *syndrome*. Based on their features, Lancereaux identified two main clinical forms. Thus, he described the “thin” diabetes (which appears in young age, and it is characterized by a speedy decrease in weight and rapid evolution towards death) and “fat” diabetes (which appears in adults in the presence of obesity, shows a hereditary nature and usually a slow and torpid evolution) [7,8]. Due to its familial nature, the second phenotype was also named as “constitutional” diabetes. All the official classifications proposed by WHO (1965, 1980, 1985 and 1998) derived from these initial observations [9]. Finally, for the two major forms of diabetes, a neutral designation of type 1 (T1D) and type 2 (T2D) diabetes was adopted.

The first important breakthrough for the elucidation of diabetes pathogenesis was represented by the autoimmune-genetic theory of T1D [3,10]. Thus, it was confirmed that diabetes is a polygenic disease and the mechanism of beta cell destruction is immune in nature. From this point, the genetic studies were planned considering that the two major phenotypes of diabetes were two different diseases. Consequently, some researchers focused on the genetics of T1D while others on the genetics of T2D. Usually, pediatric patients were selected for T1D studies [11,12], while for T2D predominantly adult and obese patients were selected. Such a “black and white” vision of diabetes phenotypes led to a tendency in highlighting mainly the differences between the two phenotypes. Moreover, the restrictive selection of patients enrolled in these genetic studies excluded almost all patients with diabetes onset between 20 and 40 years, whose separate analysis could have provided some useful information for a new thinking regarding the classification of diabetes. “Intermediary” or “secondary insulin dependent” diabetes [13], known better as “Latent Autoimmune Diabetes in Adults–LADA” [14–21], placed a *grey zone* in-between the two major phenotypes, which later proved to be associated both with classic T1D and T2D genes [11,22–27].

Genetics of T2D had a rather slow progression during the decade of candidate gene analysis, perhaps due to a not-inspired focus on the putative insulin resistance and not on the β-cell function, its true cause [28,29]^.^

The genetic landscape of the two major diabetes phenotypes included only a couple of genes at the time of the Genome Wide Association (GWA) Scan emergence, awaited with much interest and optimism. GWAs have been able to establish an extended (but only provisory) inventory of the genes associated with T1D (~50) and with T2D (~60). The number of genes associated with T1D and T2D is expected to rise in the near future [30]. However, the discovery of new genes with a significant contribution to the pathogenesis of these phenotypes is less probable.

The current genetic analysis techniques are mainly based on genotyping. Thus, genomic SNPs are tested for their association with one of the two investigated diabetes phenotypes. One major limitation of this technique resides in identifying the causal gene linked to the identified SNP, which can be placed nearby but also at some distance from that SNP [30,31]. The second constraint of this method seems to be the difficulty in describing the function of encoded proteins for many of these new genes. There is however a hope that these drawbacks will be eliminated in the future [32–35]. The third limitation is represented by a low contribution of recently identified genes to the genetic risk score of the disease [4,30,36–40]. Finally, the fourth limitation is represented by the GWA scan technique itself. Regardless of a potential higher SNP density in the future, it is hard to believe GWAS could identify some new relevant genes associated with these two phenotypes. However, a more precise localization of genes already associated with these two phenotypes is highly expected in the near future.

The current study proposes a new approach to genetic analysis as well as a complementary method to the classical GWAS analysis. Gene promoters have rarely been studied as a whole in relation to this syndrome, although their key role in the expression of genes associated with diabetes may be the root of the issue.

## Results and Discussion

Our new analysis method and the available databases enabled us to study a total of 31 promoters of genes associated with T1D (15 promoters) and T2D (16 promoters). The comparative analyses have revealed significant architectural differences between promoters of genes associated with T1D and T2D (p-Value < 0.01). Furthermore, about 16.1% of promoters belonging to T1D and over 19.3% of promoters belonging to T2D have shown some intermediary promoter patterns, suggesting a third diabetes phenotype.

### Promoters of genes classically associated with T1D

**Fig 1:**
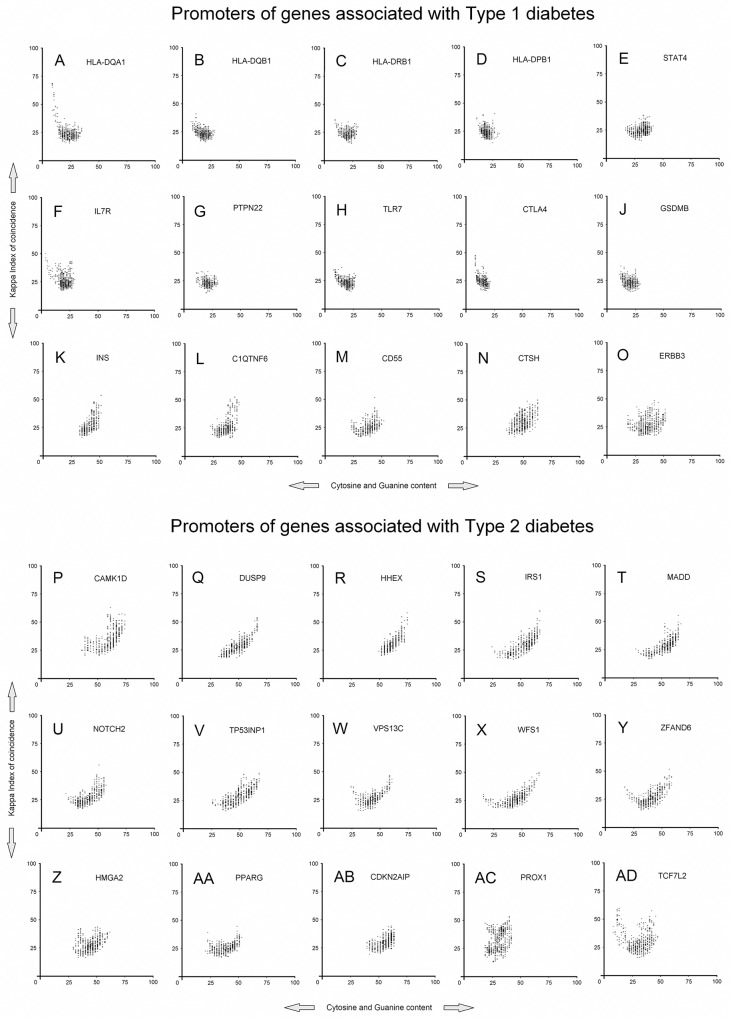
Image-based promoter patterns of genes classically associated with T1D and T2D. (A-O) promoters of genes associated with T1D, (P-AD) promoters of genes associated with T2D. Each black circle represents the center of weight of the promoter pattern.

The T1D associated genes for whom the promoter sequence was available for analysis included *PTPN22* (Protein Tyrosine Phosphatase 22), *TLR7* (Toll Like Receptor 7), *CTLA4* (Cytotoxic T Lynphocyte—assoiciated Antigen 4), *GSDMB* (Gasdermin B), *STAT4* (Signal Transducer and Activator of Transcription 4), *IL7R* (Inter-Leukin 7 Receptor), *C1QTNF6* (C1q - and Tumor Necrosis Factor—related protein 6), *CD55* (complement decay-accelerating factor), CTSH (Cathepsin H), *ERBB3* (V-ERB-B2 Avian Erythroblastic Leukemia Viral Oncogene Homolog 3), *HLA-DQA1*, *HLA-DQB1*, *HLA-DRB1*, *HLA-DPB1* (human leukocyte antigen gene family) and *INS* (Insulin gene) (Fig 1A–1O). The T1D promoters are distinguished by high (A+T)% and low Kappa IC (Kappa Index of Coincidence) values. Accordingly, T1D promoters exhibit image-based patterns characterized by centrally disposed clusters, leading to the formation of narrow shapes (Fig 1A–1O).

The lower region of these patterns contain evenly interspersed nucleotides (A,T,C,G ≈ 25%), while the upper areas show different lengths of short homopolymer tracts (poly(dA), poly(dT), poly(dC), poly(dG)) disposed in tandem in any order. The right side and the left side of these patterns are distributed in a relative 2-fold rotational symmetry. Another general characteristic of these promoters is that the average Kappa IC values are lower than 26%. The exceptions to this apparent rule are represented by *CTSH* (KIC = 31.72%), *INS* (KIC = 28.97%), and *ERBB3* (KIC = 27.35%) gene promoters, which have average Kappa IC values > 26% (S1 File). Also, *CD 55*, *CTSH* and *ERBB3* gene promoters generate slightly more relaxed patterns and show some variations of short poly(dC:dG) homopolymer tracts (Fig 1M–1O). Another interesting observation would be that promoters of *INS* and *C1QTNF6* exhibit different Kappa IC and (C+G)% average values, but have similar pattern shapes, which may suggest a direct connection in their expression (Fig 1K and 1L and S1 File). Moreover, although in accordance with all the properties described above, *HLA-DQA1* promoter also contains an atypical feature, namely additional long poly(dA:dT) homopolymer tracts (Fig 1A).

**Fig 2:**
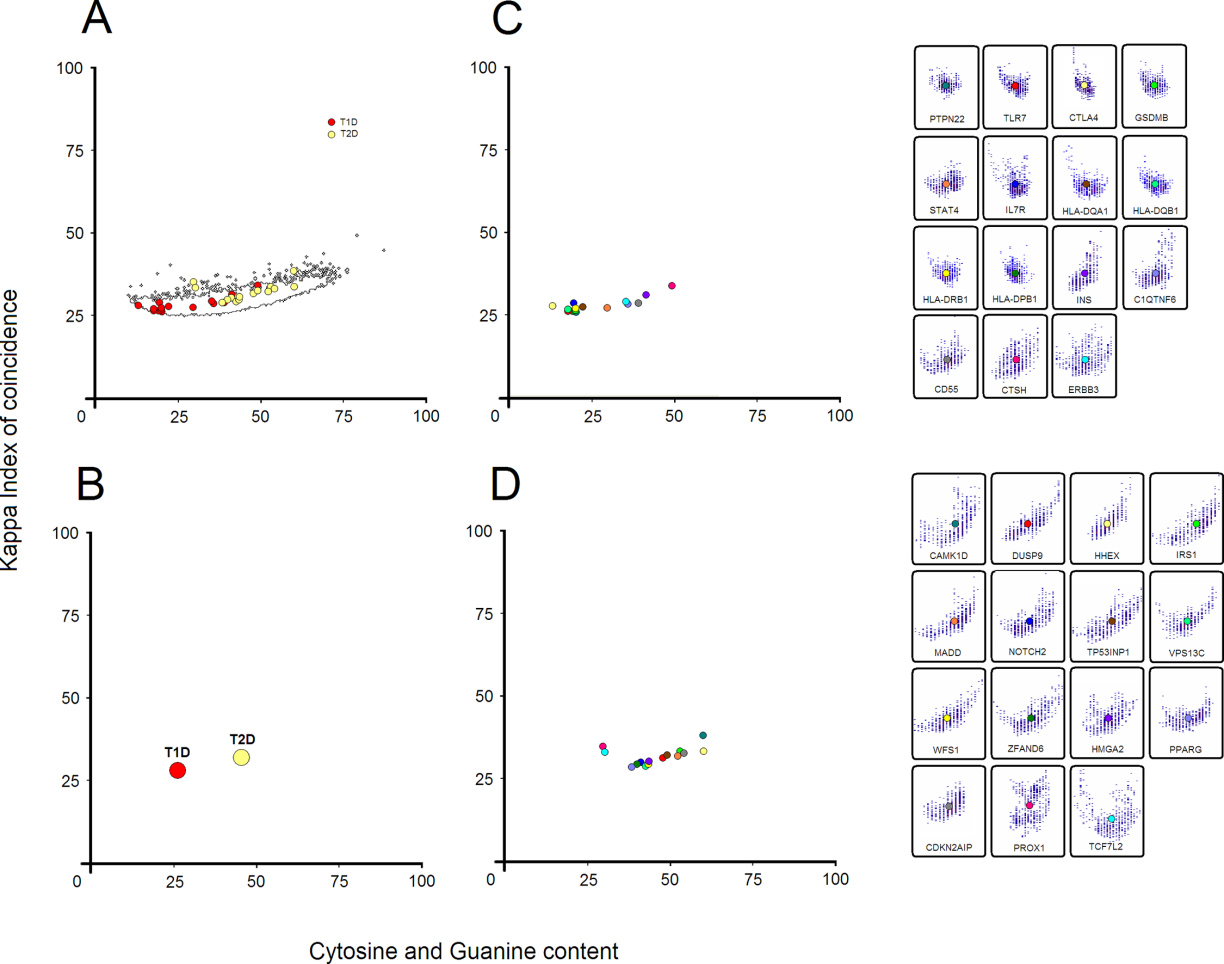
Distribution of T1D and T2D gene promoters. (A) overlapping distribution of T1D and T2D gene promoters on a genome-wide distribution of 8,515 Homo sapiens promoters, (B) mean distribution of T1D and T2D gene promoters, (C) distribution of T1D gene promoters, (D) distribution of T2D gene promoters. Each circle represents the center of weight from a promoter pattern and the circle color is associated with a corresponding gene promoter.

*HLA-DQA1* is also the only promoter that contains sequence areas with Kappa IC values higher than 50%, nevertheless, the average Kappa IC value of the entire promoter remains less than 26% (S1 File). Another feature of T1D gene promoters is represented by specific boundaries, such as (C+G)% values between 50.67% (*CTSH*) and 14.27% (*CTLA4*), and Kappa IC values between 31.72% (*CTSH*) and 23.52% (*PTPN22*). Interestingly, *CTSH* gene promoter contains the highest values, both for Kappa IC and (C+G)% (Fig 2C and S1 File). The average promoter values (Kappa IC = 25.71% and (C+G)% = 27.26) of genes associated with T1D suggests a constant lack of repetitive sequences.

### Promoters of genes classically associated with T2D

The T2D associated genes for whom the promoter sequence was available for analysis included *CAMK1*D (Calcium/Calmodulin-Dependent Protein Kinase 1—Delta), *DUSP9* (Dual-Specificity Phosphatase 9), *HHEX* (Hematopoietically Expressed Homeobox), *IRS1* (Insulin Receptor Substrate 1), *MADD* (Multiple Acyl-CoA Dehydrogenase Deficiency), *NOTCH2* (Drosophyla Homolog 2 of *NOTCH*), *TP53INP1* (Tumor Protein p53—Inducible Nuclear Protein 1), *VPS13C* (Vacuolar Protein Sorting 13 C), *WFS1* (Wolfram Syndrome 1), *ZFAND6* (Zinc Finger And Domain-Containing Protein 6), *HMGA2* (High Mobility Group At-Hook 2), *PPARG* (Peroxisome Proliferator Activated Receptor Gamma), *CDKN2A* (Cyclin-Dependent Kinase Inhibitor 2A), *PROX1* (Prospero-Related Homeobox 1) and *TCF7L2* (Transcription Factor 7 Like 2) (Fig 1P-AD).

The T2D promoters are distinguished by high (C+G)% and high Kappa IC values. T2D promoters are represented by image-based patterns containing a high percentage of C+G, a high CpG content and high Kappa IC values. The right side of the pattern is predominant while the left side is significantly less pronounced. The bi-dimensional shape of these patterns exhibits various different lengths of short poly(dC:dG) homopolymer tracts.

Unlike T1D promoters, T2D promoter patterns have a wide range in C+G content which confer a prolonged shape on the X-axis. In contrast to T1D promoters, the general characteristic of these promoters is that the average Kappa IC values are higher than 26% (Fig 2B). Overall, the promoters of genes associated with T2D exhibit a significantly higher Kappa IC average value (29.30%) than promoters of genes associated with T1D (25.71%). The T2D promoters that leap well above the genome-wide Kappa IC average belong to *PROX1*, *TCF7L2* and *CAMK1D* genes (Fig 2A).

Promoters that do not meet the common criteria of the two phenotypes belong to *PROX1* and *TCF7L2* genes. *TCF7L2* and *PROX1* gene promoters are the most interesting ones because they have a very different structure from that described for T2D so far (Fig 1AC–1AD— bottom right panels). Thus, from a total of 8,515 promoters available in EPD (The Eukaryotic Promoter Database), we found only a few image-based patterns relatively similar (ie. *SOX5* promoter) to *PROX1* promoter (Fig 1AC and S2 File). However, promoters of *TCF7L2* and *PROX1* genes comply with the (C+G)% variations found in other gene promoters associated with T2D (S1 File).

The promoter of *CD55* gene associated with T1D, and the promoters of *PPARG* and *CDKN2AIP* genes associated each with T2D phenotype, appear to have common image-based pattern shapes (Fig 1M, 1AA and 1AB). The promoters of *CD55* (Kappa IC = 26.03 and (C+G)% = 39.85) and *PPARG* (Kappa IC = 26.22 and (C+G)% = 39.04) genes are even more similar in structure since they share relatively the same Kappa IC (SD = 0.13) and (C+G)% (SD = 0.56) average values (S1 File). This similarity may suggest common mechanisms of action in their biological pathway, possibly even a direct link in their gene expression. T2D gene promoters located at the extremes are represented by (C+G)% values between 61.90% (*HHEX*) and 30.39% (*PROX1*), whereas Kappa IC values range between 35.74% (*CAMK1D*) and 26.22% (*PPARG*).

The average promoter values (KIC = 29.30% and (C+G)% = 46.91) of genes associated with T2D further show a constant presence of short poly(dC:dG) homopolymer tracts. Nevertheless, perhaps the best noticeable differences are represented by the median values shown by T1D and T2D gene promoters (Fig 2B). The median Kappa IC values presented by promoters of both phenotypes (T1D = 25.29%, T2D = 29.02%) are relatively less significant than those shown by (C+G)% median values (T1D = 20.50%, T2D = 44.79%).

### The “intermediary” phenotype of diabetes

A third, frequently encountered, phenotype of diabetes includes those patients designated along the years as “Lady-like” [41], “Type 1 1/2 diabetes” [42], “type 1.5” [43], etc. [44,45]. From a clinical point of view, this diabetes phenotype makes a smooth transition between classical “black” (T1D) and “white” (T2D) phenotypes. We designate this phenotype as Intermediary Diabetes Mellitus (IDM).

**Fig 3:**
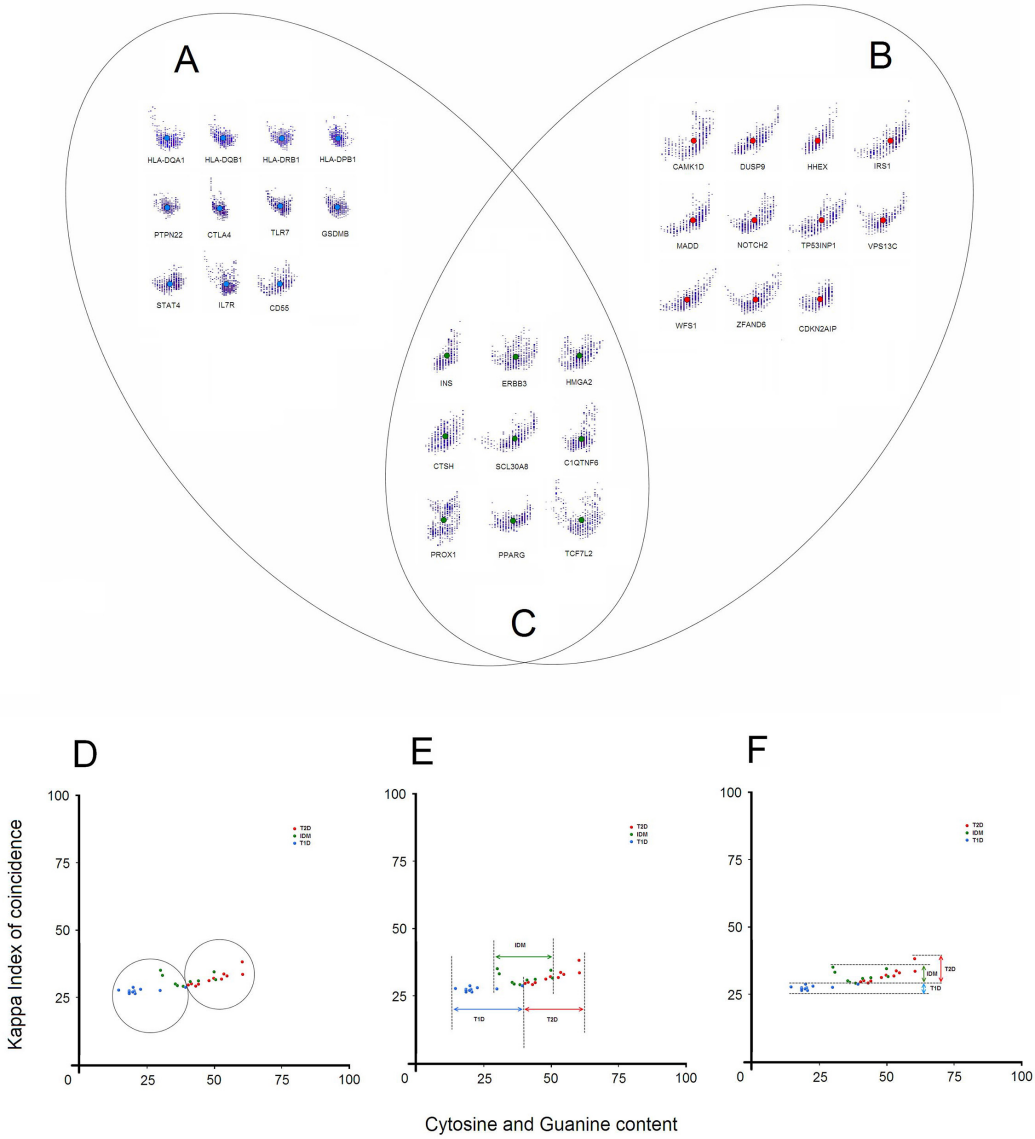
Redristribution of promoters according to T1D, IDM and T2D. (A) T1D promoters (blue dots), (B) T2D promoters (red dots) and (C) promoters from genes associated with the “intermediary” phenotype (green dots). (D) Full separation of T1D and T2D after the inclusion of the intermediary phenotype, (E) C+G values of intermediary phenotype areas overlapping equally in both T1D and T2D regions, (F) Kappa IC values of intermediary phenotype overlapping only with T2D areas.

Several genes investigated by us were reported to be associated with both T1D and T2D or have an unknown function. For instance, although *C1QTNF6* and *CTSH* genes are associated with T1D, they have an uncertain function in the context of diabetes [46]. Our analysis shows that promoter patterns of these two genes belong to an intermediate zone. Genes such as *INS* [47,48,49], *TCF7L2* [22,24,50], *SLC30A8* [23,32] or *PPARG* [12,49] have already been associated with both major phenotypes. Interestingly, promoters of these genes show the same intermediate character (Fig 3C), thus, confirming these associations. Other genes such as *ERBB3* [46,51], *HMGA2* [52,53] or *PROX1* [54–56] also have promoters that generate intermediate patterns.

**Fig 4:**
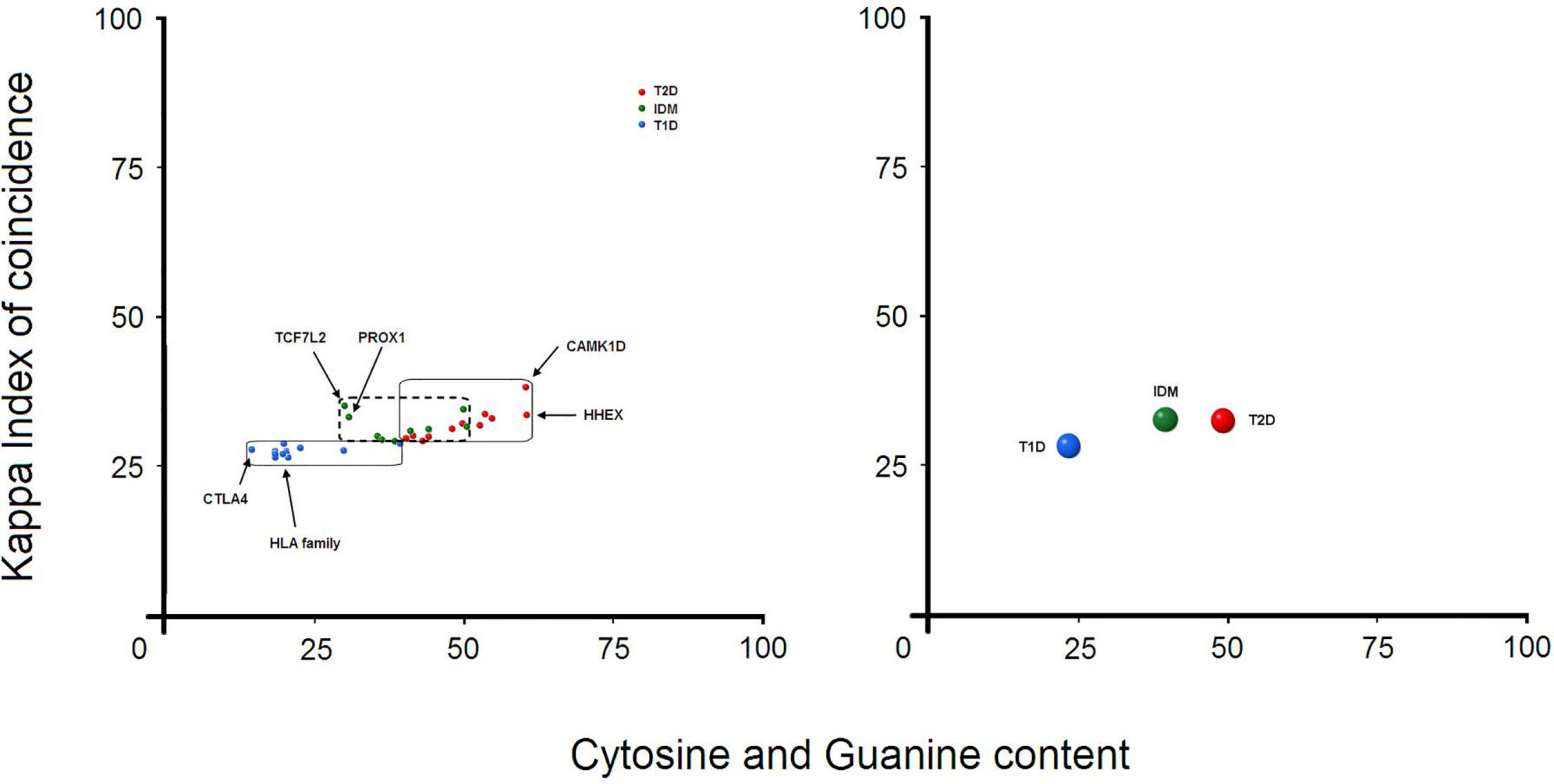
General distribution of the three phenotypes. (A) Complete separation of T1D and T2D after the inclusion of the intermediary phenotype and (B) the global distribution of the three phenotypes.

Considering the previously reported association of these genes with both phenotypes, their uncertain function and the pattern of their promoters, we can distinguish this Intermediary Diabetes Mellitus phenotype (Fig 3ABC). A general distribution of the three phenotypes shows that promoter patterns of T2D and IDM overlap (Fig 3DEF and Fig 4). However, promoter patterns of T1D do not overlap with IDM or T2D, suggesting a different pathological mechanism between T1D and IDM, but a more direct link between T2D and IDM (Fig 4A). On the other hand, extrapolating the general distribution (Fig 3DEF and Fig 2) and considering the promoter pattern shapes (Fig 3ABC), we also suggest that any gene in IDM may be a driver gene for either T1D or T2D.

### The structure of promoters and evolution

We previously reported the existence of about 10 different types of promoters in eukaryotes [57]. In general, promoters that belong to older species show lower Kappa IC and C+G values. Although unexpected, promoters of genes associated with T1D show similar structures to those that we generally have found in older eukaryotic species. Thus, a general distribution of these patterns suggests that promoters of genes associated with T1D appear to be evolutionary more conserved than those associated with T2D. This similarity in shape and angle between promoter patterns of older species and T1D is in agreement with this phenotype, which is often incompatible with life from an early age, and it was easily subjected in the past to natural selection. Furthermore, even more interesting is that T1D and T2D promoters fall into only two classes of promoters from a total of 10, namely “ATCG-compact” class (for T1D) and “CG-based” class (for T2D). In our previous studies we concluded that similar promoter patterns use similar transcription factors, which further involve different driver genes for each phenotype [57].

Interestingly, promoters of genes included in the third phenotype (IDM) have patterns that fall into several promoter classes, which may use transcription factors from both phenotypes. Furthermore, our current study suggests that gene promoters prone to methylation are those associated with T2D and IDM, but not those associated with T1D. It seems that diabetogenic influences leading to the increased prevalence of these diabetes phenotypes may act through different epigenetic mechanisms. The lack of CpG sites in the promoters of genes associated with T1D, in contrast with the high frequency of CpGs in the gene promoters associated with T2D or IDM, suggests that the mechanism of their increasing prevalence in the modern society may act through totally different pathways.

## Materials and Methods

**Fig 5:**
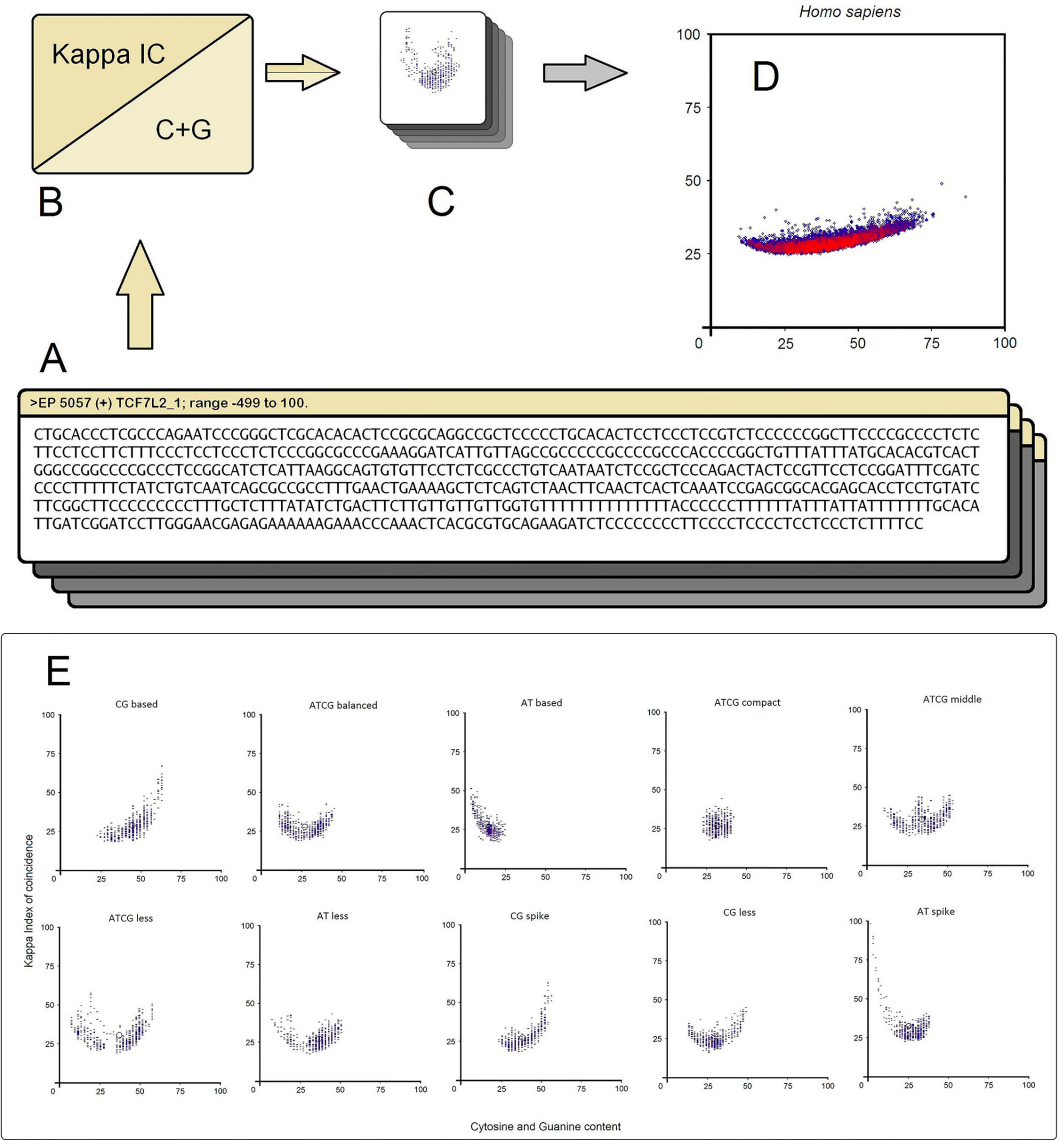
Schematic overview of the promoter analysis. (A) promoter sequences, (B) Kappa IC and (C+G)% values are extracted from each sliding window, (C) sliding window values plotted on a graph which shows a recognizable image-based pattern for each promoter sequence, (D) the center of weight of each promoter pattern plotted on a second graph in order to show the distribution of 8,515 promoters. Red color areas represent denser clusters of promoters. (E) The representative eukaryotic promoter classes are shown in the following sections: AT-based class, CG-based class, ATCG-compact class, ATCG-balanced class, ATCG-middle class, ATCG-less class, AT-less class, CG-spike class, CG-less class and ATspike class [57–59].

In our approach we used 31 promoter sequences (15 promoters from T1D and 16 promoters from T2D) obtained from Eukaryotic Promoter Database (EPD) and HomoloGene. To unravel the design principles of these promoter architectures, we have used Visual Basic to develop a software program for promoter analysis—called PromKappa (Promoter analysis by Kappa), recently published [57–59]. In brief, we used a sliding window approach (window size of 30 nucleotides (nt) and a step of 1 nt) to extract two types of values, namely Kappa IC and (C+G)%. Kappa IC values were plotted on a graph against (C+G)% values, which formed a recognizable promoter pattern for each promoter sequence (S3 File). A promoter pattern is an image that consists of 470 lines, whose coordinates have been plotted observing the two values extracted from each sliding window (Fig 5A, 5B, 5C and 5D). The shape of a pattern is composed from various sized clusters of lines on the y-axis (Fig 5D). The pattern colors range from blue to red according to the number of overlapping lines. Unlike sequence alignment algorithms, our method uses a comparison between the frequency and the nucleotide content of a promoter sequence, thereby measuring the degree of randomization of a DNA sequence [58]. The center of weight of 8,515 promoter patterns were plotted on a second graph in order to show the distribution boundaries of promoters in the human genome (Fig 5D). Next, on this distribution we superimposed the promoter locations of genes associated with T1D and T2D. For a confrontation with the promoters found in genes associated with diabetes, we show a total of 10 possible classes of gene promoters in eukaryotes (Fig 5E), found in our previous study [57].

Promoters from *INS* (insulin gene), *FTO* (fat tissue and obesity associated) and *CTLA4* (cytotoxic T lymphocyte associated antigen 4) genes, which were not found in the EPD database (composed of 8,515 Homo Sapiens promoter sequences), were extracted from HomoloGene (500 bp genomic regions upstream of the gene). Thus, promoters found in HomoloGene were introduced in the EPD data base file. Furthermore, available EPD promoters were confronted with HomoloGene genomic regions (500 bp) upstream of genes associated with T1D and T2D phenotypes in order to ensure their accuracy.

### Kappa Index of Coincidence

The Index of Coincidence (IC) principle derives from cryptography and has been used in the analysis of ciphertext. Kappa Index of Coincidence is a modified form of IC, adapted for the analysis of a single DNA sequence [57–59]. Here, Kappa IC algorithm has been used primarily as a unit of measure for the information contained in the DNA of the promoter regions.

Thus, Kappa IC is used for calculating the level of “randomization” of a DNA sequence. Kappa IC is sensitive to various degrees of sequence organization such as simple sequence repeats (SSRs) or short tandem repeats (STRs). The formula for Kappa IC is shown below, where sequences *A* and *B* have the same length *N*. Only if an *A[i]* nucleotide from sequence *A* matches the *B[i]* correspondent from sequence *B*, then ∑ is incremented by 1.

$$Kappa_{IC}=\frac{\sum_{i=1}^{N}\left[A_{i}=B_{i}\right]}{N/C}$$

The same method for measuring the Index of Coincidence has been applied for only one sequence, in which the sequence was actually compared with itself, as shown below.

function KIC(A)

T = 0

N = length(A)- 1

for u = 1 to N

B = A[u + 1] … A[N]

for i = 1 to length(B)

If A[i] = B[i] then C = C + 1

next i

T = T + (C / length(B) × 100)

C = 0

next u

IC = Round((T / N), 2)

end function

Where *N* is the length of the sliding window, *A* represents the sliding window content, *B* contains all variants of sequences generated from *A* (from *u*+1 to *N*), *C* counts the number of coincidences occurring between *B* sequence and *A* sequence and *T* counts the total number of coincidences between *B* sequences and *A* sequence.

### C+G content

We extracted *C+G* values from each sliding window considering the nucleotide frequencies from the entire promoter sequence. In the first stage, to determine the (C+G)% content for the entire (Total = TOT) promoter sequence we used the formula:

$$CG_{TOT}=\left(\frac{100}{{\left(A+T+C+G\right)}_{TOT}}\right)\times {\left(C+G\right)}_{TOT}$$

Where *CG*_*TOT*_ represents the percentage of cytosine and guanine from the promoter sequence, *(A+T+C+G)*_*TOT*_ represents the sum of the number of occurrences in the promoter sequence of A, T, C and G, and *(C+G)*_*TOT*_ represents the sum of the number of occurrences in the promoter sequence of C and G. In the next stage we used the value of *CG*_*TOT*_ to calculate the (C+G)% content from the sliding window (*sw*):

$$CG_{SW}=\left(\frac{CG_{TOT}}{{\left(A+T+C+G\right)}_{SW}}\right)\times {\left(C+G\right)}_{SW}$$

Where *CG*_*SW*_ represents the percentage of cytosine and guanine from the sliding window. These promoter patterns are relative to the percentage of C+G of the entire promoter sequence. In this regard, *CG*_*SW*_ value is relative to *CG*_*TOT*_. The expression (A+T+C+G)_TOT_ represents the sum of the number of occurrences of A, T, C and G from the sliding window sequence. *(C+G)*_*SW*_ represents the sum of the number of occurrences of C and G in the sliding window sequence.

## Conclusions

A third diabetes phenotype, known as double diabetes or 1.5 diabetes, is often observed in clinical practice. The results of our genetic analysis objectivly suports this view, showing that this *third phenotype* makes a smooth passage from T1D to T2D. It is interesting to note that Kappa IC values of IDM overlap with T2D but not with T1D. These genetic particularities may explain the difficulties of classifying some diabetic patients in the two “traditional” diabetes phenotypes. We have shown that the number of different phenotypes of diabetes is higher than two and the existence of IDM is objectively supported by our data. The third phenotype has itself two sub-phenotypes corresponding with several clinical particularities. Thus, in the near future the number of diabetes phenotypes is expected to increase, representing a strong impetus for a new classification of diabetes.

## Supporting Information

S1 File Numeric data with reference to the analysis of promoters. (XLSX)

S2 File PromKappa Diabetes software. (ZIP)

S3 File The sequences of ~ 8000 *Homo Sapiens* promoters. (ZIP)
